# Hypercortisolism in patients with cholestasis is associated with disease severity

**DOI:** 10.1186/s12876-021-02045-4

**Published:** 2021-12-07

**Authors:** Verena Theiler-Schwetz, Hansjörg Schlager, Barbara Obermayer-Pietsch, Tatjana Stojakovic, Günter Fauler, Peter Fickert, Gernot Zollner

**Affiliations:** 1 Division of Endocrinology and Diabetology, Department of Internal Medicine, Medical University of Graz, University Hospital Graz, Graz, Austria; 2 Division of Gastroenterology and Hepatology, Department of Internal Medicine, Medical University of Graz, University Hospital Graz, Graz, Austria; 3 Clinical Institute of Medical and Chemical Laboratory Diagnostics, University Hospital Graz, Graz, Austria

**Keywords:** Hypothalamic–pituitary–adrenal axis (HPA axis), Adrenal gland, Bile acids, Cholestasis, Bilirubin, Cortisol

## Abstract

**Background:**

Cholestasis might lead to an impairment of adrenal function as suggested by in vitro and in vivo data as well as by clinical findings. Bile acid and adrenal steroid metabolism not only share the receptors farnesoid X receptor (FXR) and the G protein-coupled bile acid receptor 1 (TGR5), but supraphysiological bile acid levels were found to stimulate steroidogenesis independent of FXR and TGR5. Our previous experimental findings revealed that mice fed bile acids or subjected to common bile duct ligation develop hypercortisolemia. We thus aimed to assess adrenal gland function in patients with cholestasis.

**Methods:**

Adrenal gland function was assessed in 36 patients with cholestasis and in 32 patients without cholestasis by measuring total serum cortisol, adrenocorticotropic hormone (ACTH), as well as the increase of cortisol 20 and 30 min after administration of 1 µg of ACTH. Bile acid levels and bile acid pool composition were determined by high-resolution mass spectrometry.

**Results:**

Patients with cholestasis per definition had markedly elevated levels of alkaline phosphatase (AP), bilirubin and serum bile acids. Baseline cortisol and maximum cortisol after ACTH stimulation were significantly higher in patients with cholestasis compared to controls. Increase of cortisol after ACTH stimulation and ACTH did not differ. In the cholestasis group, baseline cortisol correlated with bilirubin but not with AP, total serum bile acids and levels of conjugated and unconjugated bile acid species. Patients with duration of cholestasis < 6 months (n = 30) had significantly higher baseline cortisol levels than those with long standing cholestasis (> 6 months), together with higher bilirubin levels.

**Conclusions:**

We find no evidence of adrenal insufficiency in non-cirrhotic patients with cholestasis. In contrast, patients with cholestasis show hypercortisolism associated with disease severity as mirrored by levels of bilirubin. Lack of ACTH increase in cholestasis suggests a direct effect of cholestasis on adrenals and not on the pituitary gland. Further studies are needed to elucidate the mechanism of cortisol elevation in patients with cholestasis and its clinical significance.

## Introduction

Liver disease might impair the homeostasis of the hypothalamic–pituitary–adrenal axis (HPA axis), necessary for the regulation of stress response. Influenced by stress, physical activity, severity of illness, serum concentrations of cortisol, the hypothalamus releases corticotropin-releasing hormone (CRH), regulating the pituitary gland and stimulating the secretion of adrenocorticotropic hormone (ACTH). ACTH acts on the adrenal gland, which synthesizes and releases cortisol.

A condition termed “hepato-adrenal syndrome” has been suggested in patients with liver cirrhosis, referring to supposed adrenal insufficiency in these patients. A possible association of cholestatic liver disorders with alteration of glucocorticoid metabolism has also been suggested in past years. These assumed associations are based on several findings.

First and foremost, cholestasis—defined as a reduction or loss of bile flow caused by obstruction, disorders affecting small and large bile ducts, hereditary cholestatic syndromes or drugs—leads to hepatic and systemic retention of bilirubin and bile acids [1, 2]. Bile acids are not only essential for digestion, but are increasingly being recognized as enterohepatic hormones [3, 4]. Endocrine bile acid signalling occurs via the receptors farnesoid X receptor FXR and the G protein coupled bile acid receptor TGR5 in tissues of the enterohepatic circulation and other organs. Bile acids are synthesized in the liver from cholesterol, secreted via bile into the intestine, reabsorbed mostly in the ileum and transported back to the liver via portal blood. Bile acid transport, synthesis and metabolism as well as biliary physiology is regulated by complex mechanisms involving FXR and TGR5 [5, 6]. Both receptors are not only essential in bile, but also in glucocorticoid metabolism [7, 8]. The isoforms FXRα1 and FXRα2 are expressed both in the liver and the adrenal glands [9]. Adrenal FXR is involved in the regulation of cholesteryl ester uptake into the adrenal [10] and in synthesis of the cortisol precursor progesterone via 3-beta-hydroxysteroid-dehydrogenase type 2 (HSD3B2) in human adrenal cells in vitro [11]. Bile acids might also act via TGR5 in a cAMP/protein kinase A (PKA)-dependent fashion phosphorylating and thus activating the steroidogenic acute regulatory protein StAR. StAR is responsible for transferring cholesterol into mitochondria which is the first and rate limiting step of steroid hormone formation.

Second, data from rodent models have shown that supraphysiological bile acid levels as observed in cholestasis stimulate steroidogenesis independent of FXR and TGR5 [12]. Third, data from patients with cholestatic liver diseases undergoing surgery suggest increased mortality and clinical features suggestive of adrenal insufficiency [13, 14]. In a clinical study in women with obstructive jaundice, excretion of total cortisol metabolites such as 5b-tetrahydrocortisol and 5a-tetrahydrocortisol was reduced indicating altered cortisol clearances [15]. Furthermore, in a study comparing ACTH and cortisol levels before and after endoscopic retrograde cholangiopancreatography (ERCP) in cholestatic patients with and without tumours versus controls, levels of cortisol were significantly elevated in patients with cholestasis with tumours compared to controls over the entire observation period. Levels of ACTH were not elevated in these patients [16]. At the time of publication, circulatory cytokines were suspected to contribute to this finding [16]. Taken together, bile acids, generally elevated in cholestatic liver disease, have been suggested to play a crucial role in the development of elevated cortisol concentrations either by increasing cortisol production or by impairing cortisol clearance as outlined above. To further elucidate glucocorticoid metabolism in cholestasis, we aimed to assess adrenal function in patients with cholestasis and to compare cortisol levels with a control group of patients without cholestasis.

## Methods

Adrenal gland function was assessed in 36 patients with cholestasis (31 hospitalised, 5 outpatients) and in 32 hospitalized patients without cholestasis after obtaining written informed consent. Main outcome measures were baseline total serum cortisol and maximum total serum cortisol after stimulation with 1 µg of ACTH in both groups.

### Subjects

Eligible study participants were patients aged ≥ 18 years with a diagnosis of cholestasis. Exclusion criteria were liver cirrhosis, intake of glucocorticoids, history of pituitary or adrenal diseases or tumours, intake of aldosterone antagonists, pregnancy, and known contraindications against the injection of ACTH (such as known allergy).

Patients fulfilling the inclusion criteria and controls were recruited at the ward of the Division of Gastroenterology and Hepatology or at the Division of Endocrinology and Diabetology, Department of Internal Medicine, Medical University of Graz, Graz, Austria. Patients were asked for participation in the study and written informed consent was obtained before carrying out any study-related procedures from all subjects who agreed to participate in the study. The study was approved by the Ethics Committee of the Medical University of Graz. All study procedures were performed according to the Declaration of Helsinki and Good Clinical Practice.

### Procedures

Basal blood samples were collected in all patients and the 1 µg-ACTH test was performed in 39 patients (in 25 patients with cholestasis and in 14 controls) between 8.00 and 10.00 a.m. after an overnight fast. Total serum cortisol was measured by immunoassay (ADVIA Centaur, Siemens Healthcare Diagnostics, USA), ACTH by a chemiluminescent immunometric assay (Immulite, Siemens, USA). Aldosterone and renin were measured by chemiluminescence immunoassay (Immunodiagnostic Systems Ltd (IDS Ltd, Boldon, UK), DHEAS was measured by ELISA (Labor Diagnostika Nord GmbH, Nordhorn, Germany), as was androstenedione (DiaMetra, Milan, Italy). Relative increase of cortisol (delta cortisol) was calculated in 39 patients 20 and 30 min after administration of 1 µg-ACTH (Synacthen, Alfasigma, Italy) as a marker of relative adrenal insufficiency. Steroid hormone measurements were performed at the Endocrinology Lab Platform of the Medical University of Graz, Austria. Bile acid levels and bile acid pool composition were determined using a high-resolution mass spectrometry technique in full scan method (QExactive Orbi Trap, Thermo Scientific) as described previously [17, 18]. Biobanking of remaining blood samples was performed by freezing and storing at -80 °C until analysis.

For potential further evaluation of hypercortisolism we followed the Endocrine Society Guidelines. Accordingly, further testing for Cushing’s syndrome would have been considered only in patients with unusual features for age, patients with multiple and progressive features, particularly those who are more predictive of Cushing's syndrome or patients with adrenal incidentaloma [19]. These features were, however, not applicable to patients included in our study.

### Statistical analyses

The distribution of data was analysed by descriptive statistics and Kolmogorov–Smirnov test. Continuous data are presented as median with interquartile range or mean with standard deviation according to distribution. If necessary, skewed variables were log transformed and rechecked for normal distribution. The student’s T test or the Mann–Whitney-U test was used for comparisons of baseline characteristics between groups. All statistical procedures were performed with SPSS version 23 (SPSS Inc., Chicago, IL, USA). A *p* value < 0.05 was considered statistically significant.

## Results

68 patients were included in our analyses, 36 in the cholestasis and 32 in the control group (see Table 1). Of the 36 patients with cholestasis, 14 had pancreatic cancer or cholangiocarcinoma, one had liver metastases due to breast cancer with intrahepatic mechanical bile duct obstruction, 7 had choledocholithiasis, 6 had drug-induced cholestasis, 5 had disorders affecting small and large bile ducts (primary and secondary sclerosing cholangitis), one had autoimmune pancreatitis causing extrahepatic cholestasis, one had chronic calcifying pancreatitis, one had anastomotic stenosis after liver transplantation. Patients in the control group had esophageal stenosis, hypoglycaemia and acute kidney injury, hyponatremia, diverticulitis, myocardial infarction, pneumonia (2 patients), ketoacidosis, syncope (2 patients), gastritis, lymphoma, neuroendocrine tumour of the ileum, thoracodynia, acute pancreatitis, pancreatic cyst, uncontrolled type 2 diabetes mellitus, multiple colon polyps, hepatitis B infection, portal vein thrombosis, esophageal adenocarcinoma, esophagitis, ischemic cardiomyopathy, pancreatic carcinoma, diverticular bleeding, intraductal papillary mucinous neoplasm (3 patients), rectal carcinoma, Peutz-Jeghers syndrome, cardiorenal syndrome, dysphagia.

**Table 1:** Patient characteristics and laboratory parameters

Parameter	Cholestasis group n = 36	Control group n = 32	*p* value
Age	57.3 ± 17.0	62.6 ± 18.2	0.222*
Sex (female)	12 (33%)	12 (38%)	n.s
Bilirubin (mg/dL)	8.3 ± 6.4	0.7 ± 0.3	< 0.001*
AP (U/L)	450.5 ± 245.0	79.8 ± 24.6	< 0.001*
GGT (U/L)	549 (318–1187)	35 (19–104)	< 0.001**
AST (U/L)	112.5 (69–212)	22.5 (17–31)	< 0.001**
ALT (U/L)	171 (91–313)	23.5 (17–31)	< 0.001**
Total serum bile acids (µmol/L)	156.9 (22.9–223.4)	1.7 (1.1–3.6)	< 0.001**
CRP (mg/L)	9.8 (4.2–40.0)	2.5 (1.1–5.4)	0.009**
Aldosterone (ng/mL)	6.8 (3.7–10.5)	4.1 (3.7–11.7)	0.184**
Renin (µg/mL)	19.2 (6.9–43.6)	12.6 (5.1–29.7)	0.508**
DHEA (µg/mL)	0.5 (0.2–1.0)	0.7 (0.3–1.4)	0.143**
Androstenedione (ng/mL)	2.0 ± 1.0	3.0 ± 1.8	0.035*

AP, alkaline phosphatase; GGT, gamma-glutamyltransferase; AST, aspartate aminotransferase; ALT, alanine aminotransferase; CRP, C-reactive protein; DHEAS, dehydroepiandrosterone sulfate

*Student’s T test applied; **Mann–Whitney-U test applied

**Fig. 1:**
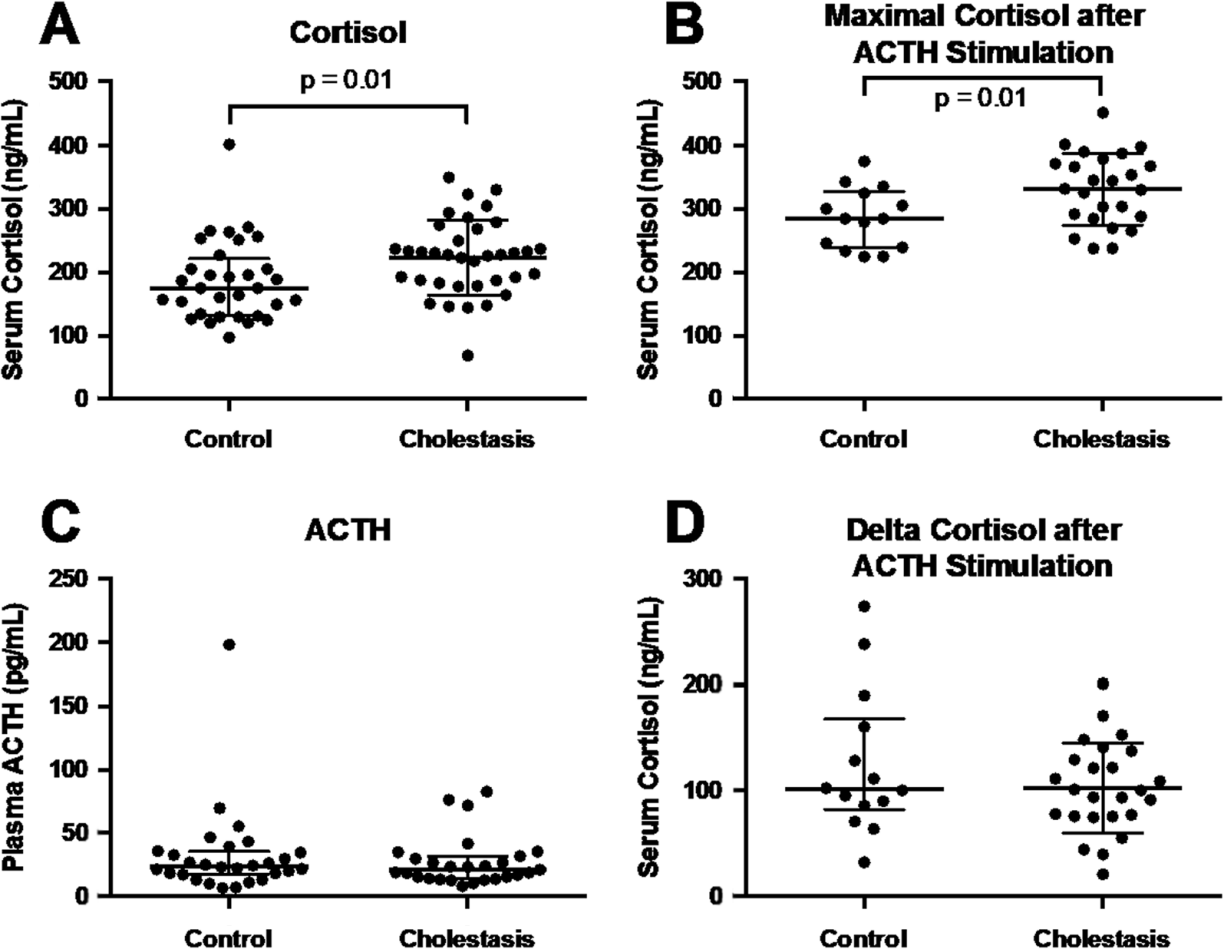
Baseline cortisol (**A**) as well as maximal cortisol levels after ACTH stimulation (**B**) are elevated in patients with cholestasis. Adrenocorticotropic hormone (ACTH) levels are not increased (**C**) indicating effects other than stimulation of the pituitary gland. Delta cortisol levels, reflecting the cortisol increase after diagnostic stimulation with ACTH, were comparable between both groups (**D**) definitively excluding adrenal insufficiency in patients with cholestasis

Patients with cholestasis per definition had markedly elevated levels of alkaline phosphatase (AP), bilirubin and serum bile acids (see Table 1). Baseline cortisol (223.2 ± 59.4 versus 186.1 ± 62.3 ng/ml, *p* = 0.016) as well as maximum cortisol after stimulation with 1 µg ACTH (Synacthen®) (331.2 ± 56.4 versus 285.9 ± 47.8 ng/ml, *p* = 0.015) were significantly higher in patients with cholestasis compared to controls (see Fig. 1A, B). Levels of ACTH, produced in the pituitary gland to stimulate cortisol synthesis and release from the adrenal glands, were not elevated in cholestatic patients (see Fig. 1C). This suggests a mechanism of cortisol increase in cholestasis other than stimulation of the pituitary gland. Delta cortisol levels, reflecting the cortisol increase after diagnostic stimulation with ACTH, were comparable between both groups (see Fig. 1D), indicating that the adrenal gland can be adequately stimulated in patients with cholestasis, thus excluding adrenal insufficiency. Levels of the other adrenal hormones measured, i.e. androstenedione, DHEAS and aldosterone, as well as renin levels were comparable between both groups (see Table 1).

**Fig. 2:**
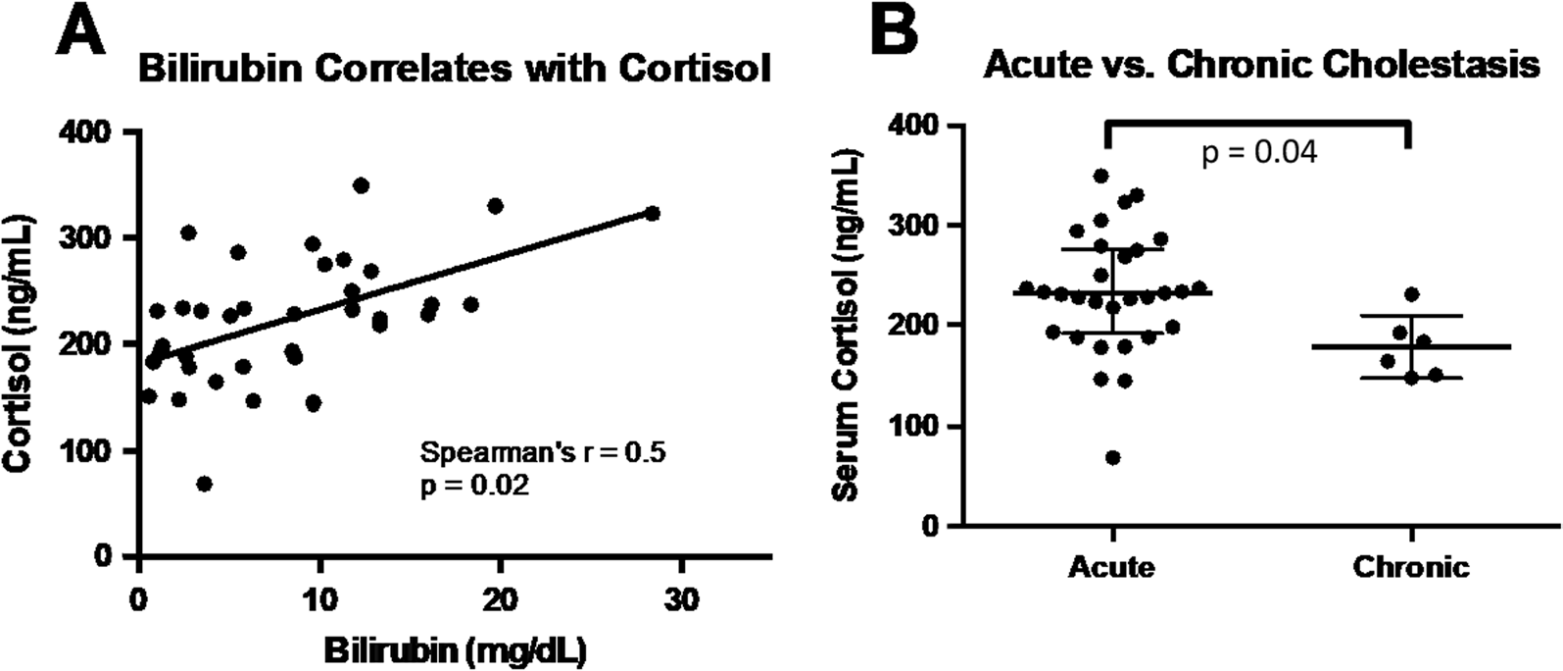
Cortisol levels directly correlated with serum bilirubin levels as a marker of disease severity (**A**). Patients with acute cholestasis (i.e. duration < 6 months) had higher serum cortisol levels than patients with longstanding disease (> 6 months)

In order to link disease severity to the extent of hypercortisolism, we analysed potential correlations between serologic markers of cholestasis and cortisol levels. Interestingly, baseline cortisol correlated with total bilirubin in patients with cholestasis (Spearman’s rho = 0.503, *p* = 0.002) (see Fig. 2A). No correlation was observed with AP and total serum bile acid levels (data not shown). Since individual bile acids might have different effects on cortisol levels, we analysed bile acid pool composition (see Table 2). However, neither levels of conjugated nor unconjugated bile acid species correlated with baseline cortisol. After the exclusion of four patients on treatment with ursodeoxycholic acid, results regarding cortisol and delta cortisol levels remained materially unchanged and serum concentrations of taurine- or glycine-conjugated ursodeoxycholic acid did not correlate with cortisol levels (data not shown). This excludes an effect of ursodeoxycholic treatment on our findings.

**Table 2:** Bile acid species

Bile acid species (µmol/L)	Cholestasis group n = 36	Control group n = 32	*p* value
UDCA	0.37 (0.23–0-78)	0.04 (0.02–0.13)	< 0.001
TUDCA	0.09 (0.04–0.26)	nd	
TCA	31.9 (3.08–64.04)	0.02 (0.00–0.07)	< 0.001
TCDCA	15.17 (2.89–26.17)	0.09 (0.03–0.19)	< 0.001
TDCA	0.16 (0.05–0.60)	nd	
TLCA	nd	nd	
GCDCA	22.41 (5.87–34.35)	0.61 (0.29–1.38)	< 0.001
GCA	55.69 (6.96–92.63)	0.14 (0.05–0.37)	< 0.001
GDCA	0.36 (0.00–1.29)	0.18 (0.03–0.58)	0.346
GUDCA	0.21 (0.08–0.40)	0.04 (0.00–0.09)	< 0.001
GLCA	nd	nd	
UDCA	nd	nd	
CA	0.04 (0.00–0.11)	0.05 (0.00–0.10)	0.824
CDCA	0.04 (0.00–0.13)	0.05 (0.00–0.11)	0.458
DCA	0.03 (0.00–0-12)	0.23 (0.01–0.40)	0.009
LCA	0.03 (0.00–0.16)	0.04 (0.00–0.06)	0.575

UDCA, ursodeoxycholic acid; TUDCA, tauroursodeoxycholic acid; TCA, taurocholic acid; TCDCA, taurochenodeoxycholic acid; TDCA, taurodeoxycholic acid; TLCA, taurolithocholic acid; GCDCA, glycochenodeoxycholic acid; GCA, glycocholic acid; GDCA, glycodeoxycholic acid; GUDCA, glycoursodeoxycholic acid; GLCA, glycolithocholic acid; CA, cholic acid; CDCA, chenodeoxycholic acid; DCA, deoxycholic acid; LCA lithocholic acid; nd, not detected

Chronic cholestasis might have different effects on adrenal function than an acute and dramatic rise of bilirubin and bile acids. In line with this hypothesis, patients with duration of cholestasis < 6 months (n = 30) had significantly higher baseline cortisol levels than those with long standing cholestasis (> 6 months, n = 6; 232.3 ± 59.9 versus 178.1 ± 31.2 ng/ml, *p* = 0.04) (see Fig. 2B). Patients with a short duration of cholestasis also had significantly higher bilirubin levels than patients with long-standing disease (9.6 ± 6.1 vs. 1.7 ± 1.4 mg/dL, *p* = 0.004) and a trend for higher bile acid levels without reaching statistical significance was observed in patients with a short duration of disease (< 6 months: 174.8 ± 138.9 vs. 94.1 ± 72.6 μmol/L, *p* = 0.180). Disease severity may thus in part explain the differences between acute and chronic cholestasis. In line with this hypothesis, baseline cortisol correlated significantly with duration of hospitalisation (Spearman’s rho = 0.331, *p* = 0.049), possibly linking severity of cholestasis to hypercortisolism.

As the presence of cancer might influence cortisol levels as previously shown [15], we compared carcinoma versus non-non-carcinoma patients. Of the 36 patients with cholestasis, 15 had a diagnosis of carcinoma, 21 had cholestasis due to other reasons. Patients with carcinoma were significantly older than those without (69 ± 10.6 vs. 49 ± 15.9 years of age). GGT, AP, bile acids, AST, ALT did not differ between groups (data not shown), bilirubin was higher in the carcinoma group (11.8 mg/dL, IQR 5.8–13.3, vs. 5.0 mg/dL, IQR 2.4–9.1, *p* = 0.039). Baseline cortisol was significantly higher in the carcinoma group (247 ± 48 vs. 206 ± 62, ng/mL *p* = 0.038), maximum cortisol did not differ between groups (349 ± 64 vs. 320 ± 49 ng/mL, *p* = 0.212). Of the patients with a short duration of cholestasis, 15 had carcinoma, 15 did not. Comparing these patients, however, no significant difference in baseline and maximum cortisol levels could be found (247 ± 48 vs. 217 ± 69 ng/ml, *p* = 0.174 for baseline cortisol, maximum cortisol 349 ± 64 vs. 325 ± 53 ng/ml, *p* = 0.352).

## Discussion

We found no evidence of adrenal insufficiency in patients with cholestasis without cirrhosis. In contrast, patients with cholestasis showed hypercortisolism associated with disease severity as mirrored by levels of bilirubin. A short duration of disease seems to have more pronounced effects on cortisol metabolism than long-standing disease. Lack of ACTH increase in cholestasis indicates a direct effect of cholestasis on adrenals and not on the pituitary gland.

We observed elevated levels of cortisol at baseline compared to controls in patients with cholestasis and comparable levels of delta cortisol after ACTH stimulation. Per definition, these findings rule out adrenal insufficiency as defined by the Endocrine Society [20]. However, as the existence of *relative* adrenal insufficiency is discussed in the context of liver disease [21–23], definitions and recommendations published by the Society of Critical Care Medicine and the European Society of Intensive Care Medicine in 2017 for the diagnosis of critical illness related corticosteroid insufficiency [24] might be more helpful, since recommendations for the evaluation of adrenal gland function in liver disease do not exist. But even considering a delta cortisol (change in baseline cortisol at 60 min) after the administration of 250 μg of ACTH of < 9 μg/dl and a random plasma cortisol of < 10 μg/dl as the cutoff, we found no evidence of adrenal insufficiency [24]. We performed 1 μg -ACTH tests, as this test might be useful in cases of special diagnostic challenges such as detection of adrenal insufficiency in critically ill patients (critical illness-related corticosteroid insufficiency), as stated in the Endocrine Society Guidelines [20]. This indicates, that in contrast to cirrhosis, patients with cholestasis do not display signs of adrenal insufficiency.

Regarding the aetiology of elevated levels of cortisol in cholestasis as compared to controls, several mechanisms seem possible. The physiologic way of increasing cortisol production via upregulation of the HPA axis, however, seems unlikely in the context of non-elevated, normal ACTH, as also observed in our study. Other potential mechanisms include increased cortisol production in the adrenal glands or reduced cortisol breakdown in the liver and the kidneys. A previous study from our group has demonstrated that supraphysiologic levels of the major human bile acid chenodeoxycholic acid (CDCA) can induce steroidogenesis in isolated mouse adrenals and in a human adrenocortical cell line [12]. This mechanism seems to be mediated by the membrane-bound sphinogose-1-phosphate receptor (S1PR2). Bile acid-activated S1PR2 led to increased expression of key enzymes in cortisol biosynthesis [12].

On the other hand, decreased cortisol degradation seems evenly likely, as various enzymes involved in bile acid breakdown in liver and kidney can be suppressed by bile acids [8]. The hepatic enzymes 5a- and 5b-reductase and 3a-hydroxysteroid dehydrogenase (3a-HSD) are responsible for irreversible inactivation of glucocorticoids [25]. Additionally, both enzymes are also involved in bile acid synthesis [26] and accumulation of bile acids leads to a suppression of 5β-reductase expression and activity. Bile acids thus cause an inhibition of hepatic glucocorticoid clearance, as shown before [15]. The second metabolic pathway of cortisol breakdown is the reversible oxidation of active cortisol into inactive cortisone via 11β- hydroxysteroid dehydrogenase (11β-HSD) type 2 in the kidney. 11β-HSD type 1, mostly expressed in the liver, primarily reduces cortisone to cortisol. Bile acids have also been shown to inhibit renal 11β-HSD2 [27, 28], thereby impairing glucocorticoid breakdown, and 11β-HSD1, preventing their reactivation [25, 29–31].

We thus hypothesize that elevated levels of cortisol in cholestasis might be caused by a reduction of cortisol breakdown and by increased cortisol production directly related to cholestasis. The fact that elevated cortisol levels correlate with severity of cholestasis could support this finding. In addition, bile acids in patients with short duration of disease are higher than in those with long-standing diseases—this, however, does not reach statistical significance, perhaps due to the low number of patients included in our study. We speculate that not only the aetiology of cholestasis (carcinoma versus other causes), nor disease duration might be solely responsible for hypercortisolism, but disease severity, i.e. the level of bile acid and bilirubin elevation, as well. One drawback regarding this hypothesis is that bile acids did not correlate with cortisol levels in our study. Most data so far linking bile acids to cortisol metabolism were obtained in well-defined experimental settings in animal models or in cell culture experiments, which cannot fully reflect complex regulatory pathways in human individuals possibly explaining our findings. The lack of a correlation between bile acids and cortisol might suggest that additional pathways are involved. We can only speculate that other factors such as bilirubin might play a mechanistic role. Bilirubin has recently been suggested to be a signalling molecule with endocrine effects. As such, bilirubin activates the aryl hydrocarbon receptor Ahr or the constitutive androstane receptor CAR and at least indirectly affects peroxisome proliferator-activated receptors (PPARs) [32].

Other limitations of our study include the relatively small number of patients included. Additionally, we did not measure relative urinary excretion of 3α,5β-tetrahydrocortisol or cortisol binding globulin [33]. Also, due to the cross-sectional nature of our study, cause-and-effect relationships cannot be elucidated. Further, we cannot rule out that the underlying disease per se—independent of the severity of the cholestasis—had an influence on cortisol levels, a problem we tried to overcome by performing subgroup analyses (cortisol levels depending on disease duration and presence or absence of carcinoma). Another limitation of the study is that we cannot definitely rule out other causes of hypercortisolism, since we did not perform dexamethasone suppression tests, targeted imaging, determine 24-h-urinary free cortisol or late-night salivary cortisol.

In summary, we cannot support the assumption of adrenal insufficiency in cholestatic liver disease which is observed in cirrhotic patients. However, we do find evidence of altered glucocorticoid metabolism in patients with cholestasis in terms of hypercortisolism, which seems to be linked to severity of cholestasis, as mirrored by the degree of bilirubin elevation. Further research needs to clarify the pathophysiology of our findings and its clinical implication for affected patients.

## Data Availability

The datasets used and/or analysed during the current study are available from the corresponding author on reasonable request.

## Abbreviations

ACTH: Adrenocorticotropic hormone
ALT: Alanine aminotransferase
AST: Aspartate aminotransferase
AP: Alkaline phosphatase
CDCA: Chenodeoxycholic acid
CRH: Corticotropin-releasing hormone
DHEAS: Dehydroepiandrosterone sulfate
ERCP: Endoscopic retrograde cholangiopancreatography
FXR: Farnesoid X receptor
GGT: Gamma-glutamyltransferase
HSD: Hydroxysteroid dehydrogenase
HPA axis: Hypothalamic–pituitary–adrenal axis
S1PR2: Sphinogose-1-phosphate receptor
TGR5: G-protein coupled bile acid receptor

## References

[1] Zollner G, Trauner M (2008). Mechanisms of cholestasis. Clin Liver Dis.

[2] Goldstein J, Levy C (2018). Novel and emerging therapies for cholestatic liver diseases. Liver Int.

[3] Houten SM, Watanabe M, Auwerx J (2006). Endocrine functions of bile acids. EMBO J.

[4] Lefebvre P, Cariou B, Lien F, Kuipers F, Staels B (2009). Role of bile acids and bile acid receptors in metabolic regulation. Physiol Rev.

[5] Deutschmann K, Reich M, Klindt C, Droge C, Spomer L, Haussinger D, Keitel V (2018). Bile acid receptors in the biliary tree: TGR5 in physiology and disease. Biochim Biophys Acta Mol Basis Dis.

[6] Chiang JYL, Ferrell JM (2019). Bile acids as metabolic regulators and nutrient sensors. Annu Rev Nutr.

[7] Trauner M, Fuchs CD, Halilbasic E, Paumgartner G (2017). New therapeutic concepts in bile acid transport and signaling for management of cholestasis. Hepatology.

[8] Theiler-Schwetz V, Zaufel A, Schlager H, Obermayer-Pietsch B, Fickert P, Zollner G (2019). Bile acids and glucocorticoid metabolism in health and disease. Biochim Biophys Acta Mol Basis Dis.

[9] Zhang Y, Kast-Woelbern HR, Edwards PA (2003). Natural structural variants of the nuclear receptor farnesoid X receptor affect transcriptional activation. J Biol Chem.

[10] Hoekstra M, van der Sluis RJ, Li Z, Oosterveer MH, Groen AK, Van Berkel TJ (2012). FXR agonist GW4064 increases plasma glucocorticoid levels in C57BL/6 mice. Mol Cell Endocrinol.

[11] Xing Y, Saner-Amigh K, Nakamura Y, Hinshelwood MM, Carr BR, Mason JI, Rainey WE (2009). The farnesoid X receptor regulates transcription of 3beta-hydroxysteroid dehydrogenase type 2 in human adrenal cells. Mol Cell Endocrinol.

[12] Liu L, Panzitt K, Racedo S, Wagner M, Platzer W, Zaufel A, Theiler-Schwetz V, Obermayer-Pietsch B, Muller H, Hofler G, Heinemann A, Zollner G, Fickert P (2019). Bile acids increase steroidogenesis in cholemic mice and induce cortisol secretion in adrenocortical H295R cells via S1PR2, ERK and SF-1. Liver Int.

[13] Zollinger RM, Williams RD (1956). Surgical aspects of jaundice. Surgery.

[14] Williams RD, Elliott DW, Zollinger RM (1960). The effect of hypotension in obstructive jaundice. Arch Surg.

[15] McNeilly AD, Macfarlane DP, O'Flaherty E, Livingstone DE, Mitic T, McConnell KM, McKenzie SM, Davies E, Reynolds RM, Thiesson HC, Skott O, Walker BR, Andrew R (2010). Bile acids modulate glucocorticoid metabolism and the hypothalamic–pituitary–adrenal axis in obstructive jaundice. J Hepatol.

[16] Zietz B, Wengler I, Messmann H, Lock G, Scholmerich J, Straub RH (2001). Early shifts of adrenal steroid synthesis before and after relief of short-term cholestasis. J Hepatol.

[17] Stojakovic T, Putz-Bankuti C, Fauler G, Scharnagl H, Wagner M, Stadlbauer V, Gurakuqi G, Stauber RE, Marz W, Trauner M (2007). Atorvastatin in patients with primary biliary cirrhosis and incomplete biochemical response to ursodeoxycholic acid. Hepatology.

[18] Amplatz B, Zohrer E, Haas C, Schaffer M, Stojakovic T, Jahnel J, Fauler G (2017). Bile acid preparation and comprehensive analysis by high performance liquid chromatography-high-resolution mass spectrometry. Clin Chim Acta.

[19] Nieman LK, Biller BM, Findling JW, Newell-Price J, Savage MO, Stewart PM, Montori VM (2008). The diagnosis of Cushing's syndrome: an Endocrine Society Clinical Practice Guideline. J Clin Endocrinol Metab.

[20] Bornstein SR, Allolio B, Arlt W, Barthel A, Don-Wauchope A, Hammer GD, Husebye ES, Merke DP, Murad MH, Stratakis CA, Torpy DJ (2016). Diagnosis and treatment of primary adrenal insufficiency: an endocrine society clinical practice guideline. J Clin Endocrinol Metab.

[21] Fede G, Spadaro L, Tomaselli T, Privitera G, Germani G, Tsochatzis E, Thomas M, Bouloux PM, Burroughs AK, Purrello F (2012). Adrenocortical dysfunction in liver disease: a systematic review. Hepatology.

[22] Fede G, Spadaro L, Tomaselli T, Privitera G, Scicali R, Vasianopoulou P, Thalassinos E, Martin N, Thomas M, Purrello F, Burroughs AK (2014). Comparison of total cortisol, free cortisol, and surrogate markers of free cortisol in diagnosis of adrenal insufficiency in patients with stable cirrhosis. Clin Gastroenterol Hepatol.

[23] Acevedo J, Fernandez J, Prado V, Silva A, Castro M, Pavesi M, Roca D, Jimenez W, Gines P, Arroyo V (2013). Relative adrenal insufficiency in decompensated cirrhosis: relationship to short-term risk of severe sepsis, hepatorenal syndrome, and death. Hepatology.

[24] Annane D, Pastores SM, Rochwerg B, Arlt W, Balk RA, Beishuizen A, Briegel J, Carcillo J, Christ-Crain M, Cooper MS, Marik PE, Umberto Meduri G, Olsen KM, Rodgers SC, Russell JA, Van den Berghe G (2017). Guidelines for the Diagnosis and Management of Critical Illness-Related Corticosteroid Insufficiency (CIRCI) in Critically Ill Patients (Part I): Society of Critical Care Medicine (SCCM) and European Society of Intensive Care Medicine (ESICM) 2017. Crit Care Med.

[25] Ackermann D, Vogt B, Escher G, Dick B, Reichen J, Frey BM, Frey FJ (1999). Inhibition of 11beta-hydroxysteroid dehydrogenase by bile acids in rats with cirrhosis. Hepatology.

[26] Danielsson H, Sjovall J (1975). Bile acid metabolism. Annu Rev Biochem.

[27] Edwards CR, Stewart PM, Burt D, Brett L, McIntyre MA, Sutanto WS, de Kloet ER, Monder C (1988). Localisation of 11 beta-hydroxysteroid dehydrogenase–tissue specific protector of the mineralocorticoid receptor. Lancet.

[28] Frey FJ (2006). Impaired 11 beta-hydroxysteroid dehydrogenase contributes to renal sodium avidity in cirrhosis: hypothesis or fact?. Hepatology.

[29] Escher G, Nawrocki A, Staub T, Vishwanath BS, Frey BM, Reichen J, Frey FJ (1998). Down-regulation of hepatic and renal 11 beta-hydroxysteroid dehydrogenase in rats with liver cirrhosis. Gastroenterology.

[30] Diederich S, Grossmann C, Hanke B, Quinkler M, Herrmann M, Bahr V, Oelkers W (2000). In the search for specific inhibitors of human 11beta-hydroxysteroid-dehydrogenases (11beta-HSDs): chenodeoxycholic acid selectively inhibits 11beta-HSD-I. Eur J Endocrinol.

[31] Morris DJ, Souness GW, Latif SA, Hardy MP, Brem AS (2004). Effect of chenodeoxycholic acid on 11beta-hydroxysteroid dehydrogenase in various target tissues. Metabolism.

[32] Vitek L (2020). Bilirubin as a signaling molecule. Med Res Rev.

[33] Verbeeten KC, Ahmet AH (2018). The role of corticosteroid-binding globulin in the evaluation of adrenal insufficiency. J Pediatr Endocrinol Metab.

